# The Taxonomic History of *Ochlerotatus* Lynch Arribálzaga, 1891 (Diptera: Culicidae)

**DOI:** 10.3390/insects12050452

**Published:** 2021-05-14

**Authors:** Lílian Ferreira de Freitas, Lyric C. Bartholomay

**Affiliations:** Department of Pathobiological Sciences, School of Veterinary Medicine, University of Wisconsin–Madison, Madison, WI 53706, USA; lyric.bartholomay@wisc.edu

**Keywords:** *Ochlerotatus*, Aedini, taxonomic history, taxonomy

## Abstract

**Simple Summary:**

Mosquitoes are an extremely diverse group of aquatic insects, distributed over all continents except Antarctica. The mosquitoes belonging to the subgenus called *Ochlerotatus*, as understood by Reinert et al., comprise a group of several species which are endemic to the Americas, some of which are important vectors of human and animal pathogens. However, this group is characteristically undefined, i.e., no sets of characters define the group, and this presents a major challenge for the understanding of the evolutionary tree of the mosquitoes. This work underscores and contextualizes the complex taxonomic history of the group, reveals the major challenges we face in order to resolve the definition of this group, and presents a path forward for its successful revision.

**Abstract:**

A review of all taxonomic actions within the subgenus *Ochlerotatus* Lynch Arribálzaga, 1891 (Diptera: Culicidae) *sensu* Reinert et al. (2008) is provided. In particular, the complex historical taxonomic treatment of the type species of this group is dissected and explained in detail. Additionally, current challenges with the definition of the subgenus and its constituents are discussed, as are the requisite steps for a successful revision of the taxon. Going forward, we conclude that a taxonomic revision of the species should include a neotype designation for *Ochlerotatus scapularis* (Rondani, 1848) from topotypical material. Additionally, we provide a review of the characters and taxa that need to be re-evaluated and well-described in order to stabilize the taxonomy of the subgenus. This effort represents a key step towards a stable nomenclature of the Tribe Aedini.

## 1. Introduction

The genus *Aedes* Meigen, 1818 (Diptera: Culicidae) has long been recognized as an unnatural assemblage [1,2]. Attempts to split the genus and resolve the classification of the Tribe Aedini [3,4,5,6,7,8,9] were met with criticism by the community of medical entomologists, public health practitioners, and mosquito control personnel who work regularly with *Aedes* species in particular. The controversy culminated in the publication of a provisional “utilitarian approach” to the Aedini nomenclature, the justification being that a more stable state of nomenclature could be achieved [10]. Such an approach fails to address the underlying problems with the taxa involved, and was met with criticism from taxonomists. One of the most contentious taxa in the works of Reinert [5], Reinert et al. [6,8,9], and Wilkerson et al. [10] is the genus *Ochlerotatus* Lynch Arribálzaga, 1891, as understood by Reinert et al. [8]. The fundamental issue with the *Ochlerotatus* is the lack of definition for the nominotypical subgenus, which stems from the polymorphic nature of the type species and from the potentially polyphyletic nature of the assemblage of species contained within. The type species, *Ochlerotatus scapularis* (Rondani, 1848), is highly polymorphic and has a vast distribution in comparison to allied taxa, being reported throughout areas east of the Andes in South America, through many islands in Central America, and in the United States in North America. The assemblage of species designated as part of the subgenus *Ochlerotatus* was only provisionally defined by Reinert et al. [8] as the Scapularis group of Arnell [11]. However, Arnell’s understanding of the group is itself ill-defined and his species descriptions contain many points of contention.

This review provides the evidence and justification for a taxonomic revision of the subgenus *Ochlerotatus* Lynch Arribálzaga as understood by Reinert et al. [8], towards the redefinition of its genus. This effort represents a key step towards a stable nomenclature of the Aedini.

## 2. Materials and Methods

We performed an exhaustive review of all literature pertaining to taxonomic actions taken on the subgenus *Ochlerotatus* Lynch Arribálzaga, 1891 (Diptera: Culicidae) *sensu* Reinert et al. [8] or any of its members, from its description in 1891 to the present. Likewise, we performed an exhaustive search for the original type specimen for the type species of the genus through communications with curators at each of the suspected locations for Rondani’s original collections. Species descriptions that were not originally in English were translated by the first author and presented in the text to highlight discrepancies and points of confusion. All discussion of anatomical characters follows the standardization of Harbach and Knight [12].

## 3. Literature Review and Discussion

### 3.1. The Description of Ochlerotatus Lynch Arribálzaga, 1891

The genus *Ochlerotatus* Lynch Arribálzaga, 1891 was described by Lynch Arribálzaga [13], who distinguished it from the similar valid genera at the time, *Culex* Linnaeus, 1758, *Janthinosoma* Lynch Arribálzaga, 1891, *Psorophora* Robineau-Desvoidy, 1827, and *Taeniorhynchus* Lynch Arribálzaga, 1891, based on the morphology of the palpi of females and the tarsal claws (of both sexes). In the newly described genus, he included *Culex albifasciatus* Macquart, 1838, synonymizing under it *Culex vittatus* Philippi, 1865. Moreover, he described a new species, *Ochlerotatus confirmatus* Lynch Arribálzaga, 1891, based on material he collected in May of 1887 near the Salado River, Buenos Aires province, Argentina, and from material sent to him by Dr. Eduardo L. Holmberg from Formosa, Chaco province, Argentina. Lynch Arribálzaga remarks on the similarity of this later species with the description of *Culex vittatus* Philippi (not Bigot), but points to the lack of an abdominal dorsal stripe on his specimens as very distinctive.

Unfortunately, Lynch Arribálzaga did not disclose the whereabouts of the specimens in his publication, and in the early 1900s he gave his entomological collections to the museum now known as the Museo Argentino de Ciencias Naturales “Bernardino Rivadavia” (MACN) [14]. O. H. Casal designated a lectotype from material found in the MACN. The lectotype is a female specimen in good condition with labels by F. Lynch Arribálzaga, identified as *Ochlerotatus confirmatus* and collected in May 1887 from Navarro (a partido in close proximity to Salado river), Buenos Aires province, Argentina (Casal in [15]).

Thereafter, in his publication on the type species of North American Diptera, in 1910, Coquillett [16] designated *Ochlerotatus confirmatus* as the type species for *Ochlerotatus*. Coquillett further expanded the genus by synonymizing under it *Protomacleaya* Theobald, 1907 and *Pseudohowardina* Theobald, 1907, for which he had previously, in 1906, already included several described genera, including: *Culicada* Felt, 1904; *Culicelsa* Felt, 1904; *Ecculex* Felt, 1904; *Protoculex* Felt, 1904; and *Pseudoculex* Dyar, 1905 [17].

### 3.2. From Culex Linnaeus, 1758 to Aedes Meigen, 1818, Genera with All-Encompassing Definitions

In 1900, Giles [18] was the first to synonymize Lynch Arribázaga’s genera (including *Heteronycha* Lynch Arribálzaga, 1891 and *Ochlerotatus* Lynch Arribálzaga, 1891) with *Culex* Linnaeus, 1758. Although he did not give a synonymy list for the genus, his treatment of species made it clear that he believed those genera belonged with *Culex* entirely. Later, in 1901, Theobald [19,20] independently utilized the same systematic treatment but gave a full synonymy list for *Culex*, along with a brief explanation for his preferred schema. However, it was Blanchard [21] who, in 1905, first published a reinterpretation of these taxa and a full explanation for the basis of his classification schema, which relied heavily on Theobald’s, focusing mostly on palpal morphology and on scale shapes. Moreover, Blanchard [21] synonymized *Ochlerotatus confirmatus* Lynch Arribálzaga, 1891 (and the treatments of *Culex confirmatus* by Giles [18,22] and Theobald [20]) under *Culex scapularis* Rondani, and provided a redescription.

Dyar and Knab [23], in 1906, discussed both Theobald’s recent classification system and Blanchard’s interpretation. They expressed concern over the validity of palpal length and scale shape as reliable characters with which to define genera. They claimed that larval characters would be of much more value for classification, and rearranged a large portion of the classification of Culicidae, with new generic definitions based on larval characters, in one of the first large revisions of the family.

One of Dyar and Knab’s [23] key contributions to the classification of Culicidae was their redefinition of the genus *Aedes* Meigen, 1818. Meigen [24] published in 1818 the description of *Aedes*, containing a single species *Aedes cinereus* Wiedemann, 1818. The description was very short but one character stood out: “*Palpi brevissimi*”. The presence of short palpi both in males and females became the mark of Meigen’s definition of *Aedes*. As short palpi in both sexes is a rare character among the Culicidae, Meigen had established already a fairly restrictive definition of the genus. Thus, the genus held very few species, and many were later removed in favor of the formation of other genera including *Haemagogus* Williston, 1896, *Uranotaenia* Lynch Arribálzaga, 1891, *Aedeomyia* Theobald, 1901, and *Wyeomyia* Theobald, 1901. When Theobald published his monograph on the Culicidae of the world, in 1901, he included only eight species under the name *Aedes*, four of which were new species [20].

Dyar and Knab [23], by strictly utilizing larval characters for taxonomic evaluation, created broadly defined generic groupings within the subfamily Culicinae. Importantly for this work, they synonymized 13 genera within *Aedes* (namely: *Ochlerotatus* Lynch Arribálzaga, 1891, *Haemagogus* Williston, 1896, *Stegomyia* Theobald, 1901, *Grabhamia* Theobald, 1903, *Howardina* Theobald, 1903, *Verrallina* Theobald, 1903, *Culicelsa* Felt, 1904, *Culicada* Felt, 1904, *Ecculex* Felt, 1904, *Protoculex* Felt, 1904, *Pseudoculex* Dyar, 1905, *Gymnometopa* Coquillett, 1906, and *Lepidoplatys* Coquillett, 1906). This broadly encompassing definition of *Aedes* is currently more representative of the tribe Aedini rather than a single genus, and their action had important repercussions on the systematics of the Aedini.

### 3.3. Early Subgeneric Divisions of Aedes Meigen, 1818

Edwards [25], in his 1917 publication, was the first to make subgeneric divisions to *Aedes sensu* Dyar and Knab [23]. Based on adult female characters, with supplemental information from male genitalia structures, he broke the genus into five subgenera (*Aedes* Meigen, 1818, *Armigeres* Theobald, 1901, *Ochlerotatus* Lych Arribálzaga, 1891, *Skusea* Theobald, 1903, and *Stegomyia* Theobald, 1901), further dividing *Aedes* (*Ochlerotatus*) into three groups (*Diceromyia* Theobald, 1911, *Finlaya* Theobald, 1903, and *Ochlerotatus* Lynch Arribálzaga, 1891), with the nominotypical group divided into three sections (*Aedimorphus* Theobald, 1903, *Ecculex* Felt, 1904, and *Ochlerotatus* Lych Arribálzaga, 1891).

In 1918, Dyar [26] reinterpreted and complemented Edward’s findings with more data from New World species. He split *Aedes* into two informal series, the Old World series and the New World series. Dyar’s [26] groupings were based exclusively on male genitalia structures, which he claimed to contain the most valuable characters for subgeneric divisions. Dyar [26] identified six groups in the Old World series, each of which he assigned a roman numeral and then tentatively named according to Edward’s subgenera, as follows: Group I—*Stegomyia* Theobald, 1901; Group II—*Skusea* Theobald, 1903; Group III—*Finlaya* Theobald, 1903; Group IV—*Armigeres* Theobald, 1901; Group V—*Ecculex* Felt, 1904, *Aedimorphus* Theobald, 1903, and *Diceromyia* Theobald, 1911; Group VI—*Aedes* Meigen, 1818. Dyar [26] applied the same system to the New World series, dividing it into five groups and tentatively naming his groups according to Edward’s subgenera or, when none was available, as he found appropriate: Group I—*Howardina* Theobald, 1903; Group II—*Gualteria* Lutz, 1904; Group III—*Taeniorhynchus* Lynch Arribálzaga, 1891; Group IV—*Ochlerotatus* Lynch Arribálzaga, 1891; Group V—*Culicada* Felt, 1904.

Later, in 1920, Dyar [27] formalized his groups as subgenera, making some modifications to his original analysis and leaving the divisions as follows:

*Howardina* Theobald, 1903 [=New World series, Group I—*Howardina*];
*Heteronycha* Lynch Arribálzaga, 1891 [=New World series, Groups IV—*Ochlerotatus* and V—*Culicada*];
*Taeniorhynchus* Lynch Arribálzaga, 1891 [=New World series, Group III—*Taeniorhynchus*];
*Finlaya* Theobald, 1903 [=Old World series, Group III—*Finlaya* and New World series, Group II—*Gualteria*];
*Stegomyia* Theobald, 1901 [=Old World series, Group I—*Stegomyia*];
*Aedes* Meigen, 1818 [=Old World series, Group VI—*Aedes*];
*Ecculex* Felt, 1904 [=Old World series, Group V—*Ecculex*, *Aedimorphus*, and *Diceromyia*].

Most notably for the work presented herein, he decided to change the association of his New World Series, Group IV, from his own understanding of Edward’s genus *Ochlerotatus* to *Heteronycha*, which became the *de facto* name of his new subgenus (*Heteronycha*) which included former Groups IV and V. Furthermore, Dyar [27] created several informal groups within the subgenus *Heteronycha*, namely: Group *pullatus*, Group *serratus*, Group *curriei*, Group *punctor*, Group *scapularis*, Group *impiger*, Group *stimulans*, Group *thibaulti*, Group *trichurus*, and Group *innuitius*.

It is important to note that in Lynch Arribálzaga’s [13] description of the genus *Heteronycha*, he included a single (newly described) species, *Heteronycha dolosa* Lynch Arribálzaga, 1891, which is the type by monotypy. Later, in 1900, Giles [18] clearly treated *Heteronycha* as synonymous with *Culex* (along with other genera described by Lynch Arribálzaga [13]). Even though he does not give a formal synonymy list for *Culex*, this is evidenced by his use of the combination *Culex dolosus* for Lynch Arribálzaga’s species. Theobald [20], in his 1901 work “A monograph of the Culicidae”, performed a major taxonomic revision of the family, also synonymizing *Heteronycha* under *Culex*. However, Theobald synonymized the name *Heteronycha dolosa* under *Culex fatigans* Wiedemann, 1828. Currently, *Culex fatigans* is a synonym of *Culex quinquefasciatus* Say, 1823, while the name *Heteronycha dolosa* is recognized under the combination *Culex dolosus* (Lynch Arribálzaga, 1891).

In 1917, Howard et al. [28] noted the morphological discrepancies between the descriptions of the male and the female in Arribálzaga’s description, observing that, although the described male was possibly a *Culex*, the female clearly belonged to *Aedes*, and so they synonymyzed *Heteronycha* with *Aedes*, creating the combination *Aedes dolosa*. Later, in 1919, Dyar [29] suggested that *Aedes* (*Ochlerotatus*) *lynchii* (Brèthes, 1910) could be a synonym of *Aedes dolosa sensu* Howard et al. [28]. If that were true, in his own words, he believed *Heteronycha* would take precedence over *Ochlerotatus*, which led to Dyar’s erroneous substitution of the name *Ochlerotatus* with *Heteronycha* in his 1920 paper on the classification of American *Aedes* [27]. The substitution was erroneous since the name *Ochlerotatus confirmatus* appears in an earlier page in Lynch Arribálzaga’s 1891 publication of both name bearing contestants for Dyar’s subgenus (namely, *Ochlerotatus confirmatus* [13] (p. 146) and *Heteronycha dolosa* [13] (p. 156), and, as such, takes position precedence as the name for the taxon (recommendation 69A.10 of the Code [30]). Afterwards, in 1921, Dyar [31] seems to believe the principle of priority would restrict the name *Heteronycha* to the synonymy of *Culex*, on the basis of Theobald’s treatment. If that were the case, he arguably failed to recognize that the earliest treatment of the name as *Culex* was actually by Giles. Even so, he was mistaken in his assertion, since none of the actions taken by Giles [18], Theobald [20], Howard et al. [28] or Dyar [27,29,31] would have any effect under consideration of the Code [30].

Dyar did, in the end, reverse his actions, and reinstituted *Aedes scapularis* syn. *Ochlerotatus confirmatus* as the name bearer for the subgenus (as opposed to *Aedes dolosa sensu* Howard et al. [28]). He also resurrected *Aedes* (*Ochlerotatus*) *lynchii* and treated the name *Heteronycha dolosa* under *Culex*, but giving the incorrect combination *Culex dolosa* [*sic*], which should be *Culex dolosus* for gender agreement, as the name *Culex* is of masculine gender and the specific epithet is a Latin adjective (*dolosa* being the feminine form of *dolosus*, meaning deceitful) (Article 31.2 of the Code [30]). O. H. Casal in [15] finally resolved the *Heteronycha* by designating a lectotype for *Heteronycha dolosa* Lynch Arribálzaga, 1891, and as the specimen is a male *Culex*, this action finally resolved the name *Heteronycha* as a synonym of *Culex*.

In the end, the type species for Dyar’s subgenus *Ochlerotatus* is *Ochlerotatus confirmatus* Lynch Arribálzaga, 1891 as the subjective synonym of *Culex scapularis* Rondani, 1848. Dyar’s Group *scapularis*, subgenus *Ochlerotatus* (later referred to as Scapularis Group [32]) therefore became a key point of reference for taxonomic changes in the subgenus, which grew significantly in the following years, as described below.

### 3.4. The Evolution of the Definition of Ochlerotatus scapularis (Rondani, 1848)

Rondani’s description of *Culex scapularis* in 1848 is fundamental to understanding the subgenus *Ochlerotatus*. Rondani used material brought by Vittore Ghiliani from Pará, Brazil, to the Museo e Instituto di Zoologia Sistemática dell’Università di Torino (TORINO), Torino, Italy, to publish a treatise on Brazilian Diptera, in which he describes *Culex scapularis*. Unfortunately, Rondani was an independent entomologist and did not leave his collections to any particular museum. Indeed, many museums acquired material from Rondani and from his publishers, Braudi and Truqui. Some believe he had left his collections in the TORINO, since Ghiliani, the original collector of the Brazilian specimens, worked there. Papavero [14] claimed that the Rondani material there was lost. Belkin et al. [15] stated that the material is “probably in Bologna”, and Arnell [11] claimed “possibly Naples”. The material from the TORINO was transferred to the Museo Regionale di Scienze Naturali, Torino, Italy, in 1978. Dr. Fulvio Giachino, the current curator for the entomology section of this museum, declared that, there are only three pins with labels written “*Culex*” present in the collection, but no specimens or any other indications (personal communication, 11 May 2016). Dr. Luca Bartolozzi, from the Florence Natural History Museum, disclosed that there is no material from Rondani housed there (personal communication, 22 November 2012). Dr. Stefano Maretti, from the Pavia Natural History Museum, informed us that only Rondani’s material of Italian origin is housed there (personal communication, 6 December 2018). The natural history museum in Milano was completely destroyed during the second World War. Dr. Alessandra Sforzi, who is currently compiling a catalog of the Rondani material, kindly informed us she had visited all of the Italian museums that collected Rondani’s material and there is a single pinned specimen that bears Rondani’s label “*Culex scapularis*”, in the Napoli museum (MZUN) [M. Zool. Num: 10866]. Unfortunately, the specimen is, in her words, very moldy, and in such bad condition that the sex cannot be determined (personal communication, 7 December 2018). Having exhausted all options in the search for the original type specimen, and given that there is no syntype in suitable conditions for a lectotype designation, and due to the polymorphic nature of the current understanding of the taxon, we conclude that a taxonomic revision of the species should include a neotype designation from topotypical material.

After Blanchard [21] synonymized, in 1905, *Ochlerotatus confirmatus* Lynch Arribálzaga, 1891, under the name *Culex scapularis* Rondani, 1848, Dyar and Knab [23] reassessed specimens previously identified by others as *Culex confirmatus* and described many new species in 1906. These include: *Aedes infirmatus* Dyar and Knab, 1906 for specimens from Baton Rouge, Louisiana, USA; *Aedes habanicus* Dyar and Knab, 1906, from Havana, Cuba (currently a subjective synonym of *Ochlerotatus tortilis* (Theobald, 1903)); and *Aedes hemisurus* Dyar and Knab, 1906 from near Spanish Town, Saint Catherine Parish, Jamaica (currently a subjective synonym of *Ochlerotatus scapularis*). In the case of the material from Jamaica, Dyar and Knab did not believe that the insular population could be conspecific with the one described from Argentina, thus they proposed a new name.

Later, in 1909, Pazos [33] added *Aedes hemisurus* Dyar and Knab, 1906 to the synonymy list of *Aedes scapularis* [34]. In the following year, Theobald [35] transferred *Aedes scapularis* to the genus *Leucomyia* Theobald, 1907, which is currently a synonym of the subgenus *Culex*. Other authors kept the species in the genus *Aedes*. In fact, Howard et al. [28] synonymized *Aedes indolescens* Dyar and Knab, 1907, and Dyar [27] later synonymized *Aedes* (*Ochlerotatus*) *camposanus* Dyar, 1918 under *Aedes scapularis*.

In successive publications, Dyar frequently revised his assessment of the Scapularis Group. In his first revision of his Scapularis Group, in 1922, Dyar [32] synonymized three taxa as subspecies, thus creating *Aedes scapularis euplocamus*, *Aedes scapularis infirmatus*, and *Aedes scapularis condolescens*. Later, in 1924, he suggested the name *Pseudohowardina* Theobald, 1907 for a subgeneric treatment of the Scapularis Group [36]. Moreover, in that same publication, Dyar [36] decided to redescribe syn. *Aedes hemisurus* as a subspecies of *Aedes scapularis*, synonymizing *Aedes indolescens* under it. He also redescribed and elevated all three taxa from his previous publication to species again (i.e., *Aedes euplocamus*, *Aedes infirmatus*, and *Aedes condolescens*).

Dyar later abandoned the subgeneric treatment of the Scapularis Group in favor of maintaining it inside the subgenus *Ochlerotatus* in his 1925 publication. He also moved *Aedes camposanus* Dyar, 1918 from synonymy with *Aedes scapularis* into synonymy with *Aedes euplocamus* Dyar and Knab, 1906 [37]. He also revoked the status of subspecies from *Aedes scapularis hemisurus*, synonymizing it with *Aedes scapularis* [38].

In the 1950s, Levi-Castillo [39,40] resurrected and redescribed *Aedes* (*Ochlerotatus*) *camposanus* Dyar, 1918. Unfortunately, he did not assign a type to this name, for which a type was not originally designated. This compounds the confusion surrounding this taxon. Similarly, in 1970, Belkin et al. [41] redescribed *Aedes* (*Ochlerotatus*) *hemisurus* Dyar and Knab, 1906 (also a typeless taxon), synonymizing *Ae*. *indolescens* Dyar and Knab, 1907 under it, in agreement with Dyar’s work from 1924 [36].

Arnell [11] performed the last major revision of the Scapularis Group in 1976. In his work, he synonymized *Aedes camposanus*, *Aedes hemisurus* (together with *Aedes indolescens*), and *Aedes* (*Ochlerotatus*) *rhyacophylus* Costa Lima, 1933 under *Aedes* (*Ochlerotatus*) *scapularis*. Later, *Aedes* (*Ochlerotatus*) *rhyacophylus* was resurrected and redescribed in 1988 by Sallum et al. [42], complete with a lectotype designation.

No taxonomic revision of *Ochlerotatus scapularis* or the Scapularis Group has been performed since Arnell published in 1976. There is understandably a significant confusion about the morphological characters that constitute the species and define the group, particularly for the type species for the subgenus, *Oc. scapularis*. It is clear that the elevation of *Ochlerotatus* Lynch Arribálzaga to genus [5] and subsequent works on the phylogeny of Aedini [6,8,9] suffer from the taxonomic uncertainty surrounding the type species of the group. This is made clear in the work by Reinert et al. from 2008 [8], where they choose to simply “define” the nominotypical subgenus for *Ochlerotatus* as the Scapularis Group *sensu* Arnell [11] as a provisional action.

### 3.5. Current Problems with the Definition of Oc. scapularis (Rondani, 1848) and Related Taxa

Below we provide detailed discussions on important characters that were used to describe each of the taxa that are or were at some point within the definition of *Ochlerotatus scapularis* as understood by Arnell [11]. For each taxon, discussion points are raised to point out discrepancies between authors’ descriptions. For the applicable taxa, translations of the original descriptions to English are presented, and the translated text is shown between double quotes (“”), with translation notes provided between square brackets ([]).

### 3.6. Ochlerotatus confirmatus Lynch Arribálzaga, 1891

Translation of the description of *Culex scapularis* Rondani, 1848 [34], from Latin:

“Brown. First antenomere yellow. Proboscis all dark. Ommatidia dark, irregularly some silvery white (Always? Also while alive?). Occiput white scaled. Thorax dorso-anteriorly white scaled, posteriorly pilose; short setae reddish, long setae brown. Pleura reddish brown, with white spots. Scutellum and mesopostnotum reddish brown. Halteres with pedicelum light brown, capitellum darker. Abdomen dark dorsally, with a pale stripe medially extending from second segment to last; at last segments more conspicuous and wider. All segments with triangular pale spots at each side. Sternites pale scaled. Legs dark anteriorly, yellowish-white posteriorly; tarsi dark. Wing membrane transparent, fourth and seventh dorsal [veins] equidistant to base of wing”.

Translation of the description of *Ochlerotatus confirmatus* Lynch Arribálzaga, 1891 [13], from Latin:

“Brown; occiput with silky-gray scales; Mesonotum with more than half of the anterior part covered in silky-silvery-goldish, posteriorly with brown falcate scales, with dark setae; dorsal part of the abdomen dark brown, slightly purplish, base of the segments silky-white, sternites silky-gray; Antennae, palpi, knees of tibiae and apex of tarsi dark. Apex of proboscis dark, base at venter lighter. Legs pale yellow. Antennae dark-brown with apexes of antenomeres with dark setae, tori and ventral base of the first antenomere scaled. Head anteriorly [clipeus?] glabrous pitch-black, posteriorly [occiput] silky-gray slightly silvery scales, brown setae, ventrally dark. Eyes in life green, after death greyish olive. Proboscis with dark scales, apex ventrally dark. Palpi dark almost black. Thorax above antral suture [scutum] densely covered in the middle with appressed silvery-grey, slightly silky-goldish, scales, posteriorly and both sides outer edges with dark falcate scales, anteriorly apparently without setae but posteriorly densely covered with long dark setae. Scutellum with dark falcate [scales?] and brown setae. Pleurae anteriorly dark, medially and posteriorly dark and silky-grey bright silvery. Wing transparent densely covered with dark scales. Halteres pale, capitellum lightly scaled. Legs pale yellow, tarsi anteriorly slightly obfuscated; knees of posterior tibial apex and tarsi diluted dark [more pale?]. Abdominal tergites dark-brown, under the light iridescent, slightly purple at margins, crosswise with a silky-white stripe, sternites with silky-gray scales”.

It is important to note here that only the patch of pale scales on the anterior part of the mesonotum and the stripe of pale scales on the terga overlap between the original descriptions of *Culex scapularis* Rondani and *Ochlerotatus confirmatus* Lynch Arribálzaga. Other currently recognized species also present a patch of pale scales on the anterior portion of the mesonotum (*Oc*. *tortilis*, *Oc*. *condolescens*, *Oc*. *euplocamus*, *Oc*. *patersoni*, *Oc*. *infirmatus*, *Oc*. *raymondi*, and *Oc*. *rhyacophilus*), and at least one other (*Oc*. *phaeonotus*) also presents (in addition to the mesonotal character) a stripe of pale scales on the terga. Yet another (*Oc*. *comitatus*) is unknown in the female form, but assumed by Arnell [11] to also present both characters. Additionally, it is clear that Arnell does not attribute much importance to the abdominal stripe character, and he says that the character is indistinct and occurs “often”, implying that it may be absent. With this consideration, the number of species that could fit the description increases considerably.

Translation of the redescription of *Culex scapularis* and synonymy of *Ochlerotatus confirmatus* within it by Blanchard in 1905, from French [21]:

“Head: falcate light cream scales medially, ochre more laterally and posteriorly, spatulate laterally; scales ochre in life [?]. Eyes: purple black and silver, encircled with spatulate ochre scales. Thorax divided into 2 zones: anteriorly, falcate scales in bright yellow, silky; posteriorly and laterally, brown scales, with 4 rows of golden brown bristles. Abdomen dark brown, with a mid-dorsal line of ochre scales, thicker at the base of the segments, clearer and wider at last one. Each segment with a white latero-basal spot. Ungues formula: 1.1.-1.1.-0.0 in the female. 2.1.-2.1.-1.1. in the male”.

Note that the ungues formula was used by culicidologists mostly in the early 1900s, to describe the state of character regarding the ventral projections of each unguis (i.e.: Presence and number of “teeth”) for the outer and inner unguis for the fore-, mid-, and hindlegs, respectively. However, little is known about the conception of this formula, and its use (in lieu of a simple description) seems to have had little to no benefit for culicidologists, as it was phased out from the literature around midcentury (personal communication, Dr. Thomas Zavortink, Dr. Ralph Harbach, 11 December 2018).

It should be noted that, for the females of *Ochlerotatus* sensu Reinert [8] the inner and outer ungues of each leg are equal. It is clear that Blanchard [21] thought the female had unequal fore- and midungues, also that they had the hindungues both untoothed (simple), but Arnell [11] considers the species as having all ungues toothed. Blanchard worked at the Faculté de Médecine de Paris. Unfortunately, we could not locate information about a voucher collection of Culicidae deposited there, thus it was impossible for us to verify the specimens he had worked with. Blanchard simply mentions a distribution including Chile, Buenos Aires (likely meaning Argentina, in general), Brazil, Guyana, and Jamaica.

The first author had access to specimens from Argentina, Brazil, and Jamaica, including the lectotype of *Oc. confirmatus* and topotypical material for *Oc. scapularis* (from Belém, Pará, Brazil). Every specimen that the first author has identified as *Oc.* aff. *scapularis* presents hindungues toothed, in accordance with Arnell [11]. Arnell describes simple hindungues (untoothed) for *Ochlerotatus condolescens*, giving its distribution as follows: Bahamas, Cuba, and Cayman Islands. The first author observed that the holotype of *Culex bracteatus* (a syn. of *Ochlerotatus tortilis*) also presents simple hindungues, and Arnell considers the Tortilis subgroup, which includes *Oc. tortilis* and *Oc. auratus*, to have hindungues toothed or simple. The distribution of *Oc. tortilis* is considered as follows: Southern Florida, Bahamas, Cuba, Grand Cayman Island, Antilles, and *Oc. auratus* occurs only in Jamaica. The scutal patterning of these species is not very similar to the one from the *Oc. scapularis* nor syn. *Culex confirmatus*, but the descriptions of the shape of the patterning which were available at the time were too vague to make a definitive identification. Therefore, it is possible that Blanchard may have analyzed specimens of these aforementioned species (perhaps from Jamaica), and not *Oc. scapularis* or *Oc. confirmatus*. However, we believe it is much more likely that Blanchard mistakenly identified a species of *Culex*, in the modern sense, as an *Ochlerotatus*, based on Theobald’s 1901 description of *Culex confirmatus* [20]. This is likely due to the fact that Theobald mentioned “fore and mid ungues equal, toothed” with no mention of the state of the hindungues. This description also seems to have caused confusion to others as well, since later, in 1910, Theobald [35], under his treatment of *Leucomyia scapularis* [=*Oc. scapularis sensu* Reinert et al. [8]] mentions that Ludlow wrote in a letter to him that she had identified a similar species to *Culex confirmatus* from Georgia, USA, with the only exception in character description being that the hind ungues were toothed. In response, Theobald clarifies that he never had seen any specimen that did not have the hind ungues toothed, and that he simply had not included the character earlier.

### 3.7. Aedes hemisurus Dyar and Knab, 1906

*Aedes hemisurus* Dyar and Knab, 1906 [23] was originally described from the larva and apparently synonymized with *Oc. scapularis* by Pazos in 1909 [33]. Pazos gives both *Ochlerotatus confirmatus* and *Aedes hemisurus* as synonyms of *Aedes scapularis sensu* Pazos, and states that the synonymy list is given by Dyar and Knab. However, those authors did not synonymize *Aedes hemisurus* in any of their publications. Pazos provides a text formatted as a reference right beside his mention of *Aedes hemisurus*, the text is as follows: “Pazos. Rev. Med. Trop., Habana (1908). t. J. p. 99”. We were unable to locate any publication or journal archive under this name, so the synonymy apparent in the literature is Pazos, 1909. Pazos [33] also provides a description, in Spanish, for the name *Aedes scapularis*. Pazos states that the description is from Theobald, thus giving the impression it is only a translation. He is misleading in this, as his description is different from Theobald’s.

Original English text from the redescription of *Culex confirmatus* by Theobald in 1901 [20]:

“Female. Head dark brown, clothed with pale creamy curved scales in the middle and with ochraceous ones at the sides and behind, and with upright ochraceous forked ones; sides with flat scales; eyes deep purplish-black and silver, with flat ochraceous scales round them; clypeus deep purplish-brown; antennae dark brown, basal joint and the greater part of the second joint testaceous; palpi short, black scaled; proboscis covered with shiny black scales. Thorax clothed in front with pale, silky, yellowish, narrow curved scales, which gradually become pure silky white about halfway across the mesonotum, the remaining part of the mesonotum darker, covered with scattered brown scales, as also are the sides, the posterior half of the mesonotum has four rows of golden-brown bristles; scutellum deep brown when viewed in one direction, ochraceous brown in the other, with creamy scales and a border of golden-brown bristles; in some specimens pale in the middle, dark at the sides; metanotum [corr.: mesopostnotum] chestnut-brown with a dull purplish tinge; pleurae chestnut-brown, with patches of white scales. Abdomen with the segments covered with deep blackish-brown scales, ground colour testaceous, this colour showing through the bases of the segments to a slight extent; down the middle of the abdomen runs a line of ochraceous scales, which are thickest at the bases of the segments, and which become lighter and spread out over the whole of the last segment; in some specimens these ochraceous scales are absent; each segment has a basal lateral patch of pure white; the hairs on the posterior borders pale brown; venter covered with creamy-yellow scales; in some specimens the apical borders of the venter have a triangular black patch on each side. Legs covered with deep brown scales with a bronzy ochraceous reflection in some lights; femora whitish beneath nearly to the apex, which is dark, coxae testaceous; hind meta tarsi [first tarsomere of hind leg] not quite so long as the hind tibiae; fore and mid ungues equal, toothed. Wings with the first sub marginal cell longer and narrower than the second posterior cell, its stem equal to about two thirds the length of the cell; stem of the second posterior cell nearly equal to the length of the cell; posterior cross-vein about its own length distant from the mid cross-vein; costa, first long vein and third long veins blackish; halteres pale with slightly fuscous knob. Length. 4.5 to 6 mm. Male. Antennae pale brownish ochraceous, with dark brown bands and brown plume-hairs; proboscis nearly as long as the palpi, dark brown; palpi covered with steel-black scales, last joint dark, like the rest of the palpus, hairs dark brown. Abdominal segments ochraceous at their bases, dark dusky-black on the apical half, which is covered with deep, dull, purplish-black scales, bases of the segments pale, partly owing to the ochraceous ground colour and partly to pale ochraceous scales, there are also a few basal white scales; from the fourth to the seventh segments are more or less triangular patches of white scales placed laterally and at the base of the segments; the last segment is covered with pule fuscous scales; claspers steel black; posterior border of the segments and the sides with long golden hairs. ungues unequal on the fore and mid legs, equal on the hind legs, similar to *C. serratus*”.

Translation of the redescription of *Aedes scapularis* and synonymy of *Aedes hemisurus* within it by Pazos in 1909, from Spanish [33]:

“Color: dark brown; thorax silvery white. Size: usually 4.5 to 6.0 mm of width [wingspan, probably]. Head: dark brown, with pale yellow scales medially and ocher laterally and posteriorly with some forked, erect and ocher in color; laterally flat scales; eyes dark, dark purple and silver; clipeus brown, dark purple; antennae dark brown; palpi with black scales; proboscis covered in shining scales. Thorax: covered anteriorly with narrow, falcate scales, silky, pale yellow, which gradually become all silky white up to the middle of the metanotum [corr.: mesonotum]; the rest of the metanotum [corr.: mesonotum] darker, covered in brown scales, tanned the same on the sides; the scutellum brownish with cream scales and dark golden setae; pleura dark brown with patches of white scales. Abdomen: dark brown with purple shine; with median line of ocher scales, denser [wider] at the base of the segments; sternites covered by cream yellow scales. Wings: with first submarginal cell [cell R_2_] longer and narrower than the second posterior [cell M_1_]; with the pedicel [vein R_2+3_] the same [size] as both margins of the cell [veins R_2_ and R_3_]; posterior transversal vein [M_3+4_] its own length distant from the median transversal vein [rm], but its location variable. Legs: covered in dark brown scales and femora whitish. Ungues: Female: all equal, not toothed. Male: anterior and median unequal, the smaller not toothed; posterior equal and not toothed”.

The most contentious aspects of these descriptions are (1) the lack of mention of the possibility for the absence of the median line of pale scales on the terga by Pazos, in contrast with Theobald, (2) the possible presence of triangular patches of dark scales on the sternites was omitted by Pazos, who describes all sternites as pale-scaled, and (3) the ungues of the female, which Pazos states are all not toothed, where Theobald says the fore- and midungues are toothed (and also clarifies later that the hindungues also is toothed [35]). Similarly, Pazos states the ungues of the male are all not toothed with the exception of the larger front ones, while Theobald says they are similar to *Culex serratus*. Theobald [20] described the ungues of *Culex serratus* as follows: “Fore and mid ungues unequal, larger one with two, smaller with one tooth; hind ungues equal, each with small thick tooth and basal swelling.” In our view, these aspects of Pazos’ description are irreconcilable with Theobald’s.

Later, in 1924, Dyar [36] recognized the taxon as a subspecies, *Aedes scapularis hemisurus*, on the basis of two male genitalia characters: 16–20 setae on basal lobe, spine [larger seta of basal lobe] stout (for *Aedes scapularis hemisurus*); 10 setae on basal lobe, spine [larger seta of basal lobe] slender (for *Aedes scapularis scapularis*). However, the same author later did not recognize this subspecific status for the taxon, in 1928, and slightly changed his view of the characters of the male genitalia of *Aedes scapularis*, stating: “[…] basal lobe with four or five setae and other minute ones adjacent to a large spine with swollen base” [38].

In 1970, Belkin et al. [41] recognized specific rank to *Ae. hemisurus* and provided a redescription of all life stages. These authors emphasized that the main difference of this species from *Aedes scapularis* is the lack of a retrorse process on the claspette filament, and the presence of fewer setae on the distal part of the basal lobe; this is a departure from the previous interpretation by Dyar, i.e., that *Aedes scapularis* has fewer setae on the basal lobe.

Arnell [11] synonymized the taxon again within *Aedes scapularis* in 1976, together with two more species (*Aedes camposanus* and *Aedes rhyacophilus*), in addition to his keeping of the synonyms given by earlier authors. He also gives a broader description of the male genitalia of this new concept of *Ae. scapularis*, such that it became indistinguishable from *Aedes phaeonotus* Arnell, 1976, a species of his own description. *Aedes phaeonotus* is distinguishable from *Ae. scapularis* only in the female based on the color of the anterior patch of pale scales of the mesonotum, which should be yellowish tan for *Aedes phaeonotus* and white for *Aedes scapularis*. This is not a useful character, for the following reasons: (1) Scale bleaching is a problem for identification of stored specimens, and (2) the first author has observed extreme variability in the coloration of the mesonotal patch of pale scales from mosquitoes from one single collection event, including freshly collected mosquitoes where the median portion of the patch was white and the edges were golden yellow. This in itself does not preclude the possibility that one species may emerge with white scaling and the other develops the character by sun exposure during life. However, it would be impossible to tell which of these phenomena has happened for any given specimen. Arnell restricted the occurrence of *Aedes phaeonotus* to Grenada [11], but the first author has observed mosquitoes from Brazil which have yellow anterior patches of mesonotal scales, which is in accordance with Arnell’s descriptions [11]. Arnell was the first to restrict the coloration of the mesonotal anterior scale patch to white [11].

### 3.8. Aedes indolescens Dyar and Knab, 1907

*Aedes indolescens* Dyar and Knab, 1907 [43] was described from the adult female and not differentiated in the description from *Aedes scapularis*, and later synonymized with it in 1917 by Howard et al. [28] and subsequently with *Aedes hemisurus* by Dyar [36], in 1924, with no indication of the justification for the decision.

### 3.9. Aedes camposanus Dyar, 1918

*Aedes camposanus* Dyar, 1918 [26] is described both in the female and the male, with the main difference from other species described for the male genitalia, i.e., the claspette filament without a retrorse expansion.

### 3.10. Aedes rhyacophilus Costa Lima, 1933

*Aedes rhyacophilus* Costa Lima, 1933 [44] was described in the adult female and male, including male genitalia, and larva, with the main differences presented for the male genitalia, the most important of which was the absence of the apical lobe of the gonocoxite. Arnell [11] synonymized the species in 1976 under *Aedes scapularis* but did not mention the possibility for lack of an apical lobe. Sallum et al. [42], resurrected and redescribed the species in 1988, clearly showing various differences from Arnell’s description of *Aedes scapularis*, including but not limited to: The presence of posterior scutal fossal setae and achrostical setae, and the absence of a median stripe of pale scaling on the terga in the adult female; the lack of apical lobe of the gonocoxite in the adult male genitalia.

## 4. Summary of Findings

Based on the aspects of characters historically presented in the literature and the experience of the first author with these characters, we compiled a summary of findings that will be central to a review of the *Ochlerotatus*.

The presence or absence of the occipital dorsolateral spots of dark scaling was treated as a variable feature in the entirety of the Scapularis group by Arnell [11]. However, we have reason to believe that the state of absence of this character matches with other sets that form a more restricted group. We contend that the subgenus *Ochlerotatus* should include only species for which the character is absent.

The achrostical setae development has been treated too broadly and is not consistently observed, as evidenced in the treatment of *Ochlerotatus ryacophilus* by Arnell as a synonym of *Ochlerotatus scapularis*. He describes *Oc. scapularis* as not having achrostical setae, but *Oc. rhyacophilus* has them developed in a complete row [42]. This feature is therefore important, and has been clearly misrepresented in Arnell’s descriptions, which served as a basis for analysis of the *Ochlerotatus* and its definition by Reinert et al. [8].

The dorsocentral setae, similarly, were considered as present posteriorly and absent anteriorly for the group. However, the first author has found that the anterior row is not absent in every species included in the group by Arnell, revealing yet another of Arnell’s oversights. Furthermore, the first author found scutal fossal setae presence and distribution to be of taxonomical interest for this group, but the character is only mentioned as present or absent by Arnell. Even then, many of his descriptions are in disagreement with the previous descriptions of species or synonyms he presented. This is clear in the example of *Oc. rhyacophilus* and specimens of *Oc*. aff. *scapularis* from southern Brazil, both of which present posterior scutal fossal setae [42] (unpublished data). Arnell [11] considers the posterior scutal fossal setae absent in *Oc. scapularis*, yet both morphotypes would fall within *Oc. scapularis* in his work, both by taxonomic action (*Oc*. *rhyacophilus* is considered a synonym of *Oc*. *scapularis*) and by the characters provided in his key to species.

The white scaling patterns in the tibiae and tarsi also have been found to be different than reported for the Scapularis subgroup of Arnell. The taxonomic significance of the presence of simple claws is also to be discussed, as it is not addressed by Arnell, who synonymized *Oc. bracteatus* within *Oc. tortilis*. There are also other reasons to believe this synonymy is incorrect. The scaling patterns of the abdomen have been treated by Arnell as important for species differentiation, however, the description of *Oc. scapularis* features all ranges of variation of said character, which renders this character both unusable and incorrect.

Arnell overemphasizes the shape of the mesonotal pattern of scales, making various concessions to several character variations considered important in other mosquito species.

Additionally, the first author has found variations in male genitalia characters, especially the shape of the apical lobe and the aedeagus, between the populations of mosquitoes identified to *Ochlerotatus scapularis sensu* Arnel from Belém, Pará, Brazil, and Santa Catarina, Brazil, as well as Chaco, Argentina.

We hope to clarify the variation on the abdominal scaling pattern and to discuss its relative importance within the group, as well as variations in the morphology of the male genitalia, in a future publication.

## 5. Conclusions

In conclusion, this review presents an exhaustive description of the taxonomic history of *Ochlerotatus* (*Ochlerotatus*) and delineates the amount and complexity of change inflicted on this group from the original description by Rondani in 1848 to present. We depict all of the taxonomic actions surrounding this group in the Supplemental Material. It is important to note that this complicated history reflects the change in understanding of taxonomy itself, and later, of the underlying evolutionary forces driving speciation. This history also reflects the efforts of taxonomists to capture such understandings in classification schemes, as well as their disagreements on the relative importance of different pieces of evidence that constitute the hypothesis-species and higher taxa.

In particular, this work reveals major disagreements with the treatments of several characters that were understood as fundamental for the group classification by Arnell [11], and delineates how they have ultimately prevented definition for the group in the work of Reinert et al. [8]. The data show the need for a redescription of *Ochlerotatus scapularis* (Rondani), in order to understand and describe the variations of the subgenus *Ochlerotatus*. Towards that end, we also present the exhaustive search for the type specimen, culminating in the finding that no original specimen in good condition for redescription exists. Therefore, fixation of a neotype for the species *Ochlerotatus scapularis* is of paramount importance. Moreover, we provide a path of characters and taxa that need to be re-evaluated and well-described in order to stabilize the taxonomy of the subgenus.

This review provides the first step in the process to revise the genus *Ochlerotatus* from a confusing, long, and fraught taxonomic legacy. We contend that taking steps to filling this knowledge gap is key to stabilizing the classification of Aedini, the largest tribe of Culicidae, in order to provide improved understanding of its phylogenetic relationships, both internally and within the Culicinae.

## Supplementary Materials

The following are available online at https://www.mdpi.com/article/10.3390/insects12050452/s1. Supplementary file S1: The taxonomic history of the names within *Ochlerotatus* (*Ochlerotatus*) Lynch Arribálzaga, 1891. This material is a visual representation of the historical taxonomic actions taken on every name associated with the subgenus *Ochlerotatus* at some point in time. It is subdivided into six panels for ease of visualization.

## References

[1] Edwards F.W. (1932). Diptera Fam. Culicidae. Genera Insectorum.

[2] Harbach R.E., Kitching I.J. (1998). Phylogeny and Classification of the Culicidae (Diptera). Syst. Entomol..

[3] Reinert J.F. (1999). Restoration of *Verrallina* to Generic Rank in Tribe Aedini (Diptera: Culicidae) and Descriptions of the Genus and Three Included Subgenera. Contrib. Am. Entomol. Inst..

[4] Reinert J.F. (2000). Restoration of *Ayurakitia* to Generic Rank in Tribe Aedini and a Revised Definition of the Genus. J. Am. Mosq. Control Assoc..

[5] Reinert J.F. (2000). New Classification for the Composite Genus *Aedes* (Diptera: Culicidae: Aedini), Elevation of Subgenus *Ochlerotatus* to Generic Rank, Reclassification of the Other Subgenera, and Notes on Certain Subgenera and Species. J. Am. Mosq. Control Assoc..

[6] Reinert J.F., Harbach R.E., Kitching I.J. (2004). Phylogeny and Classifcation of Aedini (Diptera: Culicidae), Based on Morphological Characters of All Life Stages. Zool. J. Linn. Soc..

[7] Reinert J.F., Harbach R.E., Kitching I.J. (2006). Phylogeny and Classification of *Finlaya* and Allied Taxa (Diptera: Culicidae: Aedini) Based on Morphological Data from All Life Stages. Zool. J. Linn. Soc..

[8] Reinert J.F., Harbach R.E., Kitching I.J. (2008). Phylogeny and Classifcation of *Ochlerotatus* and Allied Taxa (Diptera: Culicidae: Aedini) Based on Morphological Data from All Life Stages. Zool. J. Linn. Soc..

[9] Reinert J.F., Harbach R.E., Kitching I.J. (2009). Phylogeny and Classifcation of Aedini (Diptera: Culicidae). Zool. J. Linn. Soc..

[10] Wilkerson R.C., Linton Y.-M., Fonseca D.M., Schultz T.R., Price D.C., Strickman D.A. (2015). Making Mosquito Taxonomy Useful: A Stable Classification of Tribe Aedini That Balances Utility with Current Knowledge of Evolutionary Relationships. PLoS ONE.

[11] Arnell J.H. (1976). Mosquito Studies (Diptera: Culicidae) XXXIII: A Revision of The Scapularis Group of *Aedes* (*Ochlerotatus*). Contrib. Am. Entomol. Inst..

[12] Harbach R.E., Knight K.L. (1980). Taxonomists’ Glossary of Mosquito Anatomy.

[13] Lynch Arribálzaga F. (1891). Dipterologia Argentina. Rev. Mus. Plata.

[14] Papavero N. (1973). Essays on the History of Neotropical Dipterology: With Special Reference to Collectors (1750–1905).

[15] Belkin J.N., Schick R.X., Heinemann S.J. (1968). Mosquito Studies (Diptera, Culicidae) XI: Mosquitoes Originally Described from Argentina, Bolivia, Chile, Paraguay, Peru, and Uruguay. Contrib. Am. Entomol. Inst..

[16] Coquillett D.W. (1910). The Type-Species of the North American Genera of Diptera. Proc. United States Natl. Mus..

[17] Coquillett D.W. (1906). A Classification of the Mosquitoes of North and Middle America.

[18] Giles G.M.J. (1900). A Handbook of the Gnats or Mosquitoes: Giving the Anatomy and Life History of the Culicidæ.

[19] Theobald F.V. (1901). A Monograph of the Culicidae, or Mosquitoes: Mainly Compiled from the Collections Received at the British Museum from Various Parts of the World in Connection with the Investigation into the Cause of Malaria Conducted by the Colonial Office and the Royal.

[20] Theobald F.V. (1901). A Monograph of the Culicidae, or Mosquitoes: Mainly Compiled from the Collections Received at the British Museum from Various Parts of the World in Connection with the Investigation into the Cause of Malaria Conducted by the Colonial Office and the Royal.

[21] Blanchard R., de Rudeval F.R. (1905). Les Moustiques: Histoire Naturelle et Medicále.

[22] Giles G.M.J. (1902). A Handbook of the Gnats or Mosquitoes: Giving the Anatomy and Life History of the Culicidæ Together with Descriptions of All Species Noticed up to the Present Date.

[23] Dyar H.G., Knab F. (1906). The Larvae of Culicidae Classified as Independent Organisms. J. N. Y. Entomol. Soc..

[24] Meigen J.W. (1818). Systematische Beschreibung Der Bekannten Europäischen Zweiflügeligen Insekten.

[25] Edwards F.W. (1917). Notes on Culicidae, with Descriptions of New Species. Bull. Entomol. Res..

[26] Dyar H.G. (1918). The Male Genitalia of *Aedes* as Indicative of Natural Affinities (Diptera, Culicidae). Insecutor Inscitiae Menstruus.

[27] Dyar H.G. (1920). The Classification of American *Aedes* (Diptera, Culicidæ). Insecutor Inscitiae Menstruus.

[28] Howard L.O., Dyar H.G., Knab F. (1917). The Mosquitoes of North and Central America and the West Indies.

[29] Dyar H.G. (1919). A Note on Argentine Mosquitoes (Diptera, Culicidæ). Insecutor Inscitiae Menstruus.

[30] International Commission on Zoological Nomenclature (ICZN) (2000). International Code of Zoological Nomenclature.

[31] Dyar H.G. (1921). The Mosquitoes of Argentina (Diptera, Culicidæ). Insecutor Inscitiae Menstruus.

[32] Dyar H.G. (1922). The American *Aedes* of the Scapularis Group (Diptera, Culicidæ). Insecutor Inscitiae Menstruus.

[33] Pazos J.H. (1909). Contribucion Al Estudio de Los Mosquitos de Cuba. Secr. Sanid. Benefic. República Cuba.

[34] Rondani C. (1848). Esame Di Varie Specie d’insetti Ditteri Brasiliani. Stud. Entomol..

[35] Theobald F.V. (1910). A Monograph of the Culicidae, or Mosquitoes: Mainly Compiled from the Collections Received at the British Museum from Various Parts of the World in Connection with the Investigation into the Cause of Malaria Conducted by the Colonial Office and the Royal.

[36] Dyar H.G. (1924). Note on the American *Aedes* of the Scapularis Group (Diptera, Culicidæ). Insecutor Inscitiae Menstruus.

[37] Dyar H.G. (1925). The Mosquitoes of Panama (Diptera, Culicidæ). Insecutor Inscitiae Menstruus.

[38] Dyar H.G. (1928). The Mosquitoes of the Americas.

[39] Levi-Castillo R. (1951). Nota Taxonómica Sobre La Especie Ecuatoriana *Aedes* (*Ochlerotatus*) *camposanus* Dyar, 1918 (Dipt. Culicidae). Rev. Entomol..

[40] Levi-Castillo R. (1952). Redescription of *Aedes* (*Ochlerotatus*) *camposanus* Dyar (1918) as a Valid Species Found in the Coastal Plain of Ecuador. Pac. Sci..

[41] Belkin J.N., Heinemann S.J., Page W.A. (1970). The Culicidae of Jamaica (Mosquito Studies. XXI). Contrib. Am. Entomol. Inst..

[42] Sallum M.A.M., Uramoto K., Forattini O.P. (1988). Redescription, and Resurrection from Synonymy, of *Aedes* (*Ochlerotatus*) *rhyachophilus* Costa Lima, 1933. Mem. Inst. Oswaldo Cruz.

[43] Dyar H.G., Knab F. (1907). Descriptions of Some American Mosquitoes. J. N. Y. Entomol. Soc..

[44] Costa Lima A. (1933). Sobre Um Novo *Aëdes* (*Ochlerotatus*) Do Brasil. Mem. Inst. Oswaldo Cruz.

