# Supplementary material for: The Taxonomic History of Ochlerotatus Lynch Arribálzaga, 1891 (Diptera: Culicidae)
Source: Insects. 2021 May 14;12(5):452. doi: 10.3390/insects12050452 (PMC8156232; doi:10.3390/insects12050452)
Supplement: Supplementary file 1 [file insects-12-00452-s001.zip › insects-1187833-SI.pdf]

Figure S1. The taxonomic history of the names within *Ochlerotatus* (*Ochlerotatus*) Lynch Arribálzaga, 1891: the orignal combinations *Aedes infirmatus* Dyar & Knab, 1906; *Aedes condolescens* Dyar & Knab, 1907; *Aedes euplocamus* Dyar & Knab, 1906; *Aedes* (*Ochlerotatus*) *camposanus* Dyar, 1918; *Culex scapularis* Rondani, 1848; *Ochlerotatus confirmatus* Lynch Arribálzaga, 1891; *Aedes hemisurus* Dyar & Knab, 1906; *Aedes indolescens* Dyar & Knab, 1907 and *Aedes* (*Ochlerotatus*) *rhyacophilus* Costa Lima, 1933.

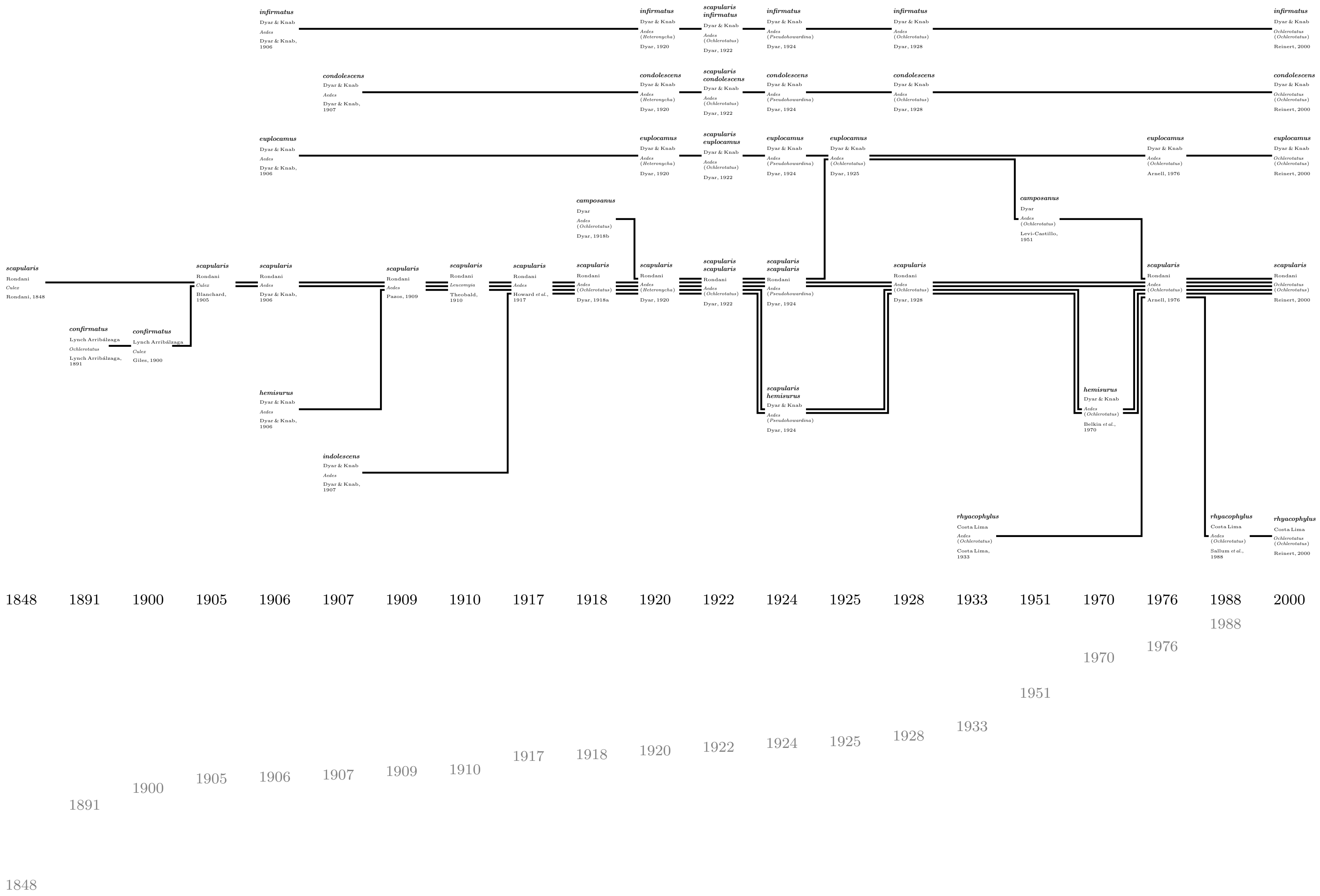

Legend: for each node, the specific and subspecific (when applicable) epithets are given in bold on top, followed by the author; afterwards follows the generic and subgeneric (when applicable) treatments at the time (given by the position of the node); at the bottom the reference is given for the treatment at the time; the lines represent the shifting understanding of each original combination; the x-axis gives the years for the publications, with the shadows (in gray) representing the distance of that year from the most recent year in the figure (*i.e.* 2000).

Figure S2. . The taxonomic history of the names within *Ochlerotatus* (*Ochlerotatus*) Lynch Arribálzaga, 1891: the orignal combinations *Aedes* (*Ochlerotatus*) *meprai* Martínez & Prosen, 1953; *Aedes* (*Ochlerotatus*) *traversus* Dyar, 1925; *Aedes argentescens* Dyar & Knab, 1908; *Aedes cuneatus* Dyar & Knab, 1908; *Aedes angustivittatus* Dyar & Knab, 1907; *Culex inconspicuus* Grossbeck, 1904 and *Culex trivittatus* Coquillett, 1902.

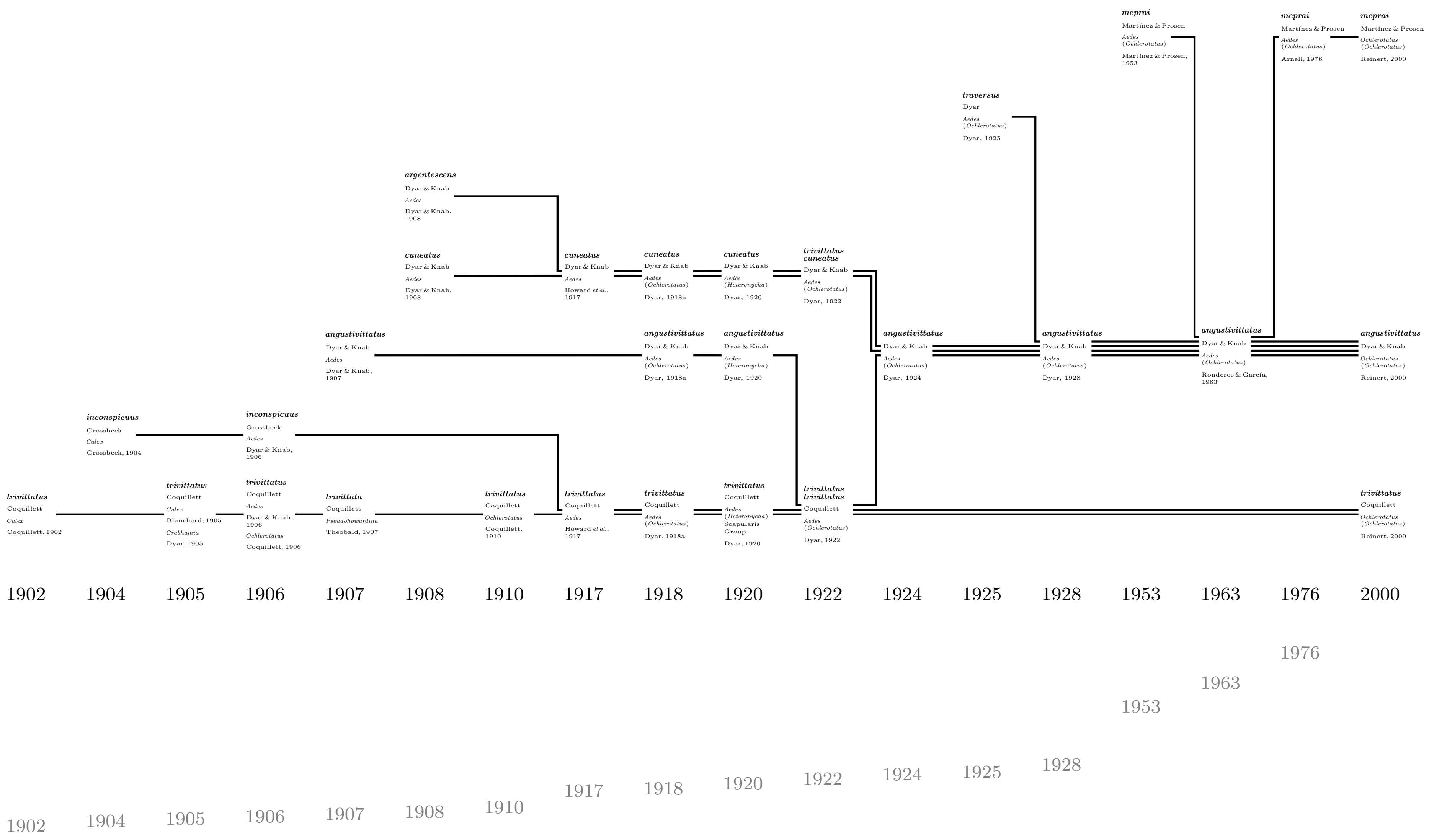

Legend: for each node, the specific and subspecific (when applicable) epithets are given in bold on top, followed by the author; afterwards follows the generic and subgeneric (when applicable) treatments at the time (given by the position of the node); at the bottom the reference is given for the treatment at the time; the lines represent the shifting understanding of each original combination; the x-axis gives the years for the publications, with the shadows (in gray) representing the distance of that year from the most recent year in the figure (*i.e.* 2000).

Figure S3. The taxonomic history of the names within *Ochlerotatus* (*Ochlerotatus*) Lynch Arribálzaga, 1891: the orignal combinations *Culex lynchii* Brèthes, 1911; *Culex tapinops* Brèthes, 1917; *Aedes* (*Ochlerotatus*) *iguazu* Shannon & Del Ponte, 1928; *Culex crinifer* Theobald, 1903 and *Aedes* (*Ochlerotatus*) *patersoni* Shannon & Del Ponte, 1927; the taxonomic history of the original combination *Heteronycha dolosa* Lynch Arribálzaga, 1891 that intersects the one of this group is also shown.

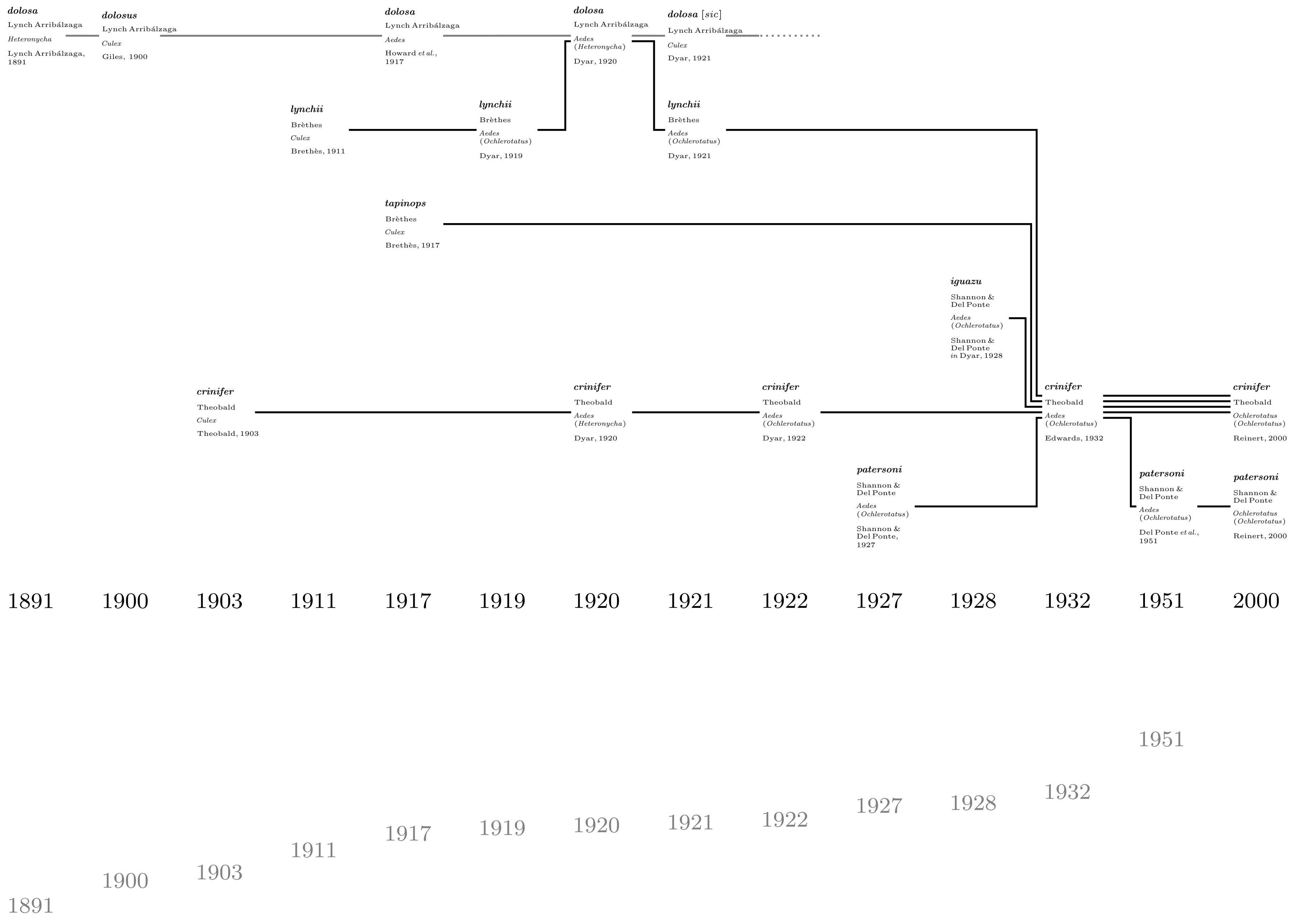

Legend: for each node, the specific and subspecific (when applicable) epithets are given in bold on top, followed by the author; afterwards follows the generic and subgeneric (when applicable) treatments at the time (given by the position of the node); at the bottom the reference is given for the treatment at the time; the lines represent the shifting understanding of each original combination (a grey line represents a name that does not pertain to this revision, the taxonomic history it shares with this group is presented in the figure, but not beyond that); the x-axis gives the years for the publications, with the shadows (in gray) representing the distance of that year from the most recent year in the figure (*i.e.* 2000).

Figure S4. The taxonomic history of the names within *Ochlerotatus* (*Ochlerotatus*) Lynch Arribálzaga, 1891: the orignal combinations *Aedes* (*Ochlerotatus*) *tortilis virginensis* Dyar, 1922; *Aedes plutocraticus* Dyar & Knab, 1907; *Aedes balteatus* Dyar & Knab, 1907; *Aedes habanicus* Dyar & Knab, 1906; *Aedes bracteatus* Coquillett, 1906a; *Culex tortilis* Theobald, 1903 and *Aedes auratus* Grabham, 1906.

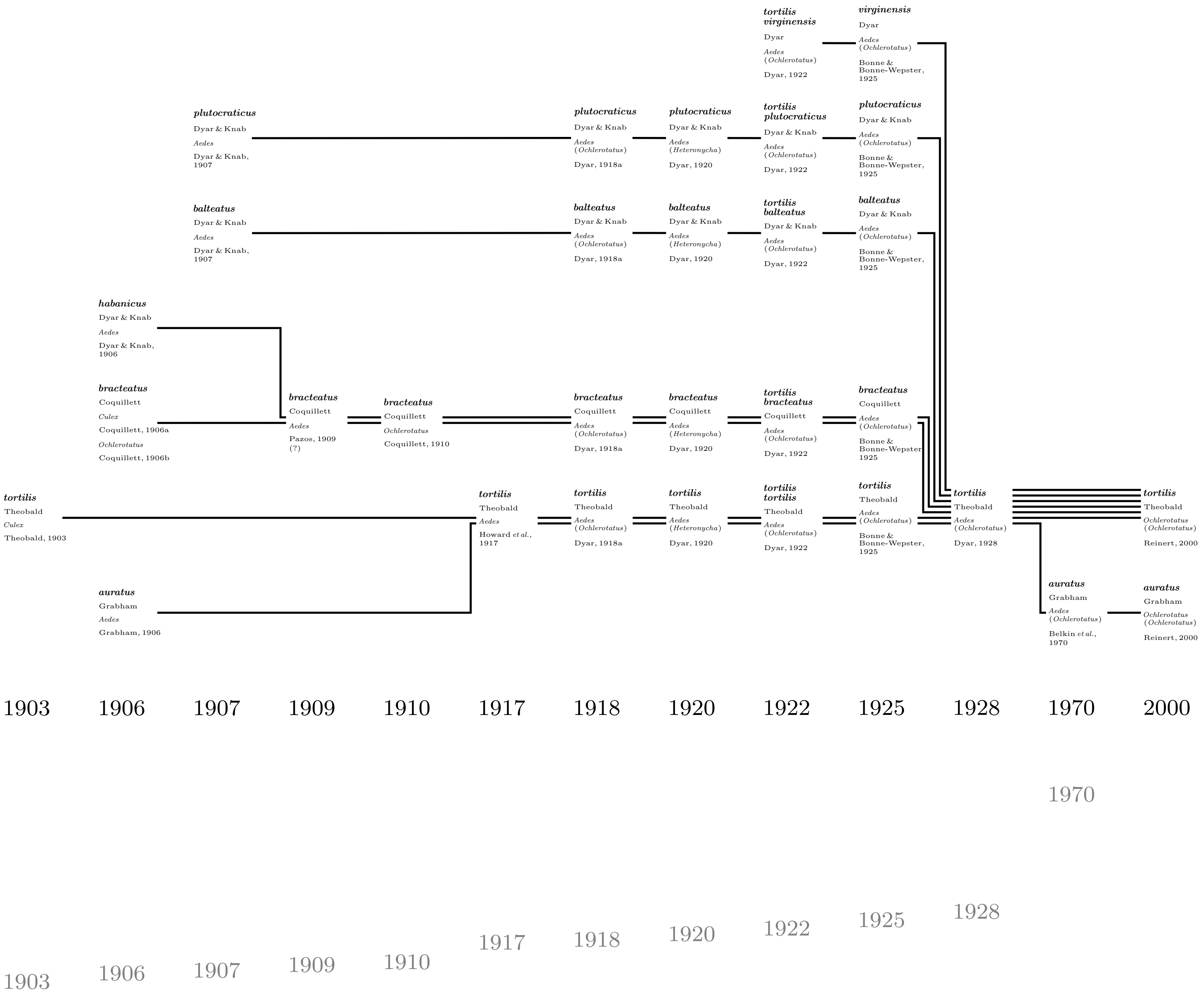

Legend: for each node, the specific and subspecific (when applicable) epithets are given in bold on top, followed by the author; afterwards follows the generic and subgeneric (when applicable) treatments at the time (given by the position of the node); at the bottom the reference is given for the treatment at the time; the lines represent the shifting understanding of each original combination; the x-axis gives the years for the publications, with the shadows (in gray) representing the distance of that year from the most recent year in the figure (*i.e.* 2000).

Figure S5 . The taxonomic history of the names within *Ochlerotatus* (*Ochlerotatus*) Lynch Arribálzaga, 1891: the orignal combinations *Aedes* (*Ochlerotatus*) *raymondi* Del Ponte *et al.*, 1951; *Aedes* (*Ochlerotatus*) *keyensis* Buren, 1947; *Aedes* (*Taeniorhynchus*) *thelcter* Dyar, 1918 and *Aedes obturbator* Dyar & Knab, 1907; the taxonomic history of the original combinations *Aedes milleri* Dyar, 1922 and *Culex bilineatus* Theobald, 1903 that intersects the one of this group is also shown.

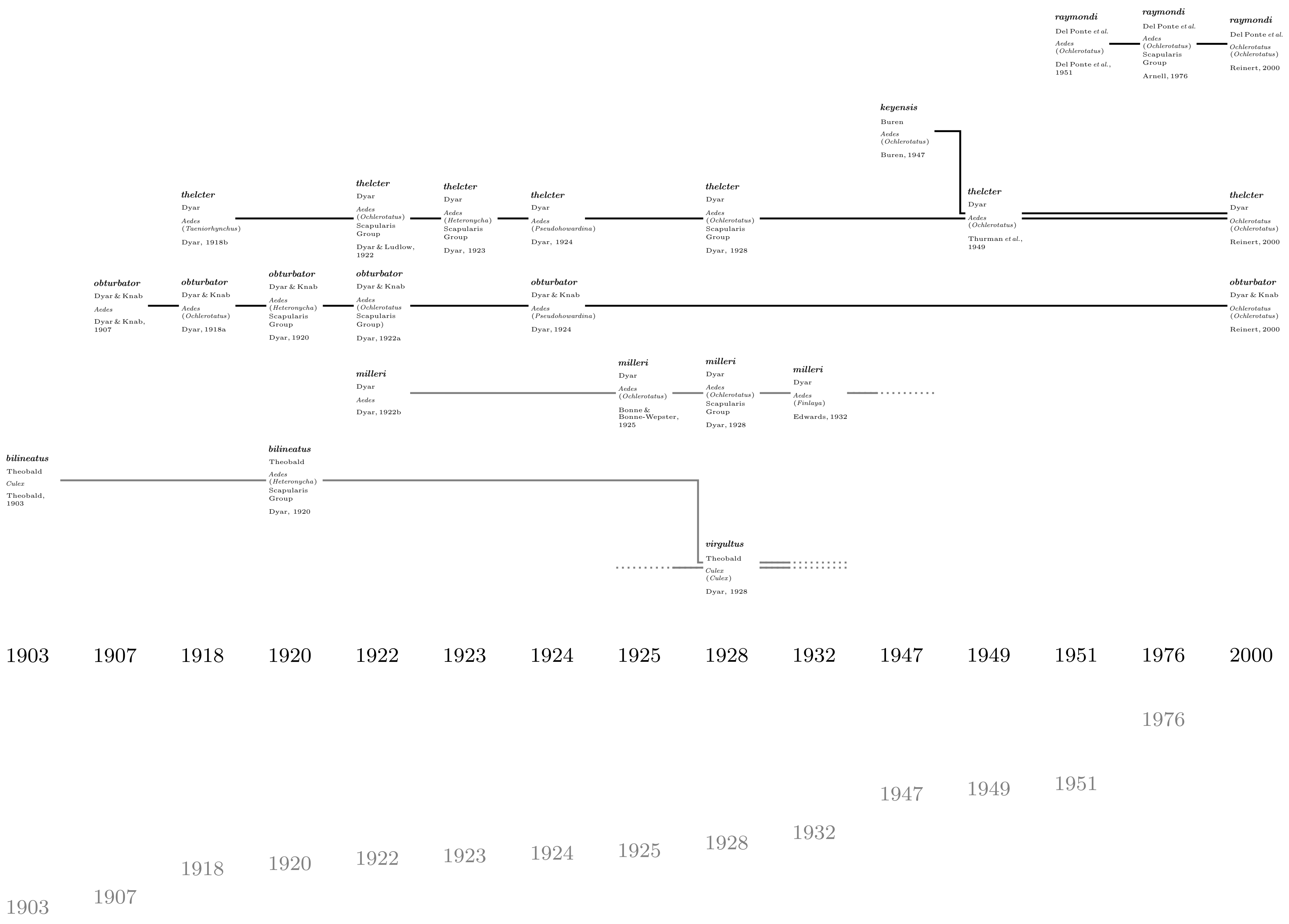

Legend: for each node, the specific and subspecific (when applicable) epithets are given in bold on top, followed by the author; afterwards follows the generic and subgeneric (when applicable) treatments at the time (given by the position of the node); at the bottom the reference is given for the treatment at the time; the lines represent the shifting understanding of each original combination (a grey line represents a name that does not pertain to this revision, the taxonomic history it shares with this group is presented in the figure, but not beyond that); the x-axis gives the years for the publications, with the shadows (in gray) representing the distance of that year from the most recent year in the figure (*i.e.* 2000).

Figure S6. The taxonomic history of the names within *Ochlerotatus* (*Ochlerotatus*) Lynch Arribálzaga, 1891: the orignal combinations *Aedes* (*Ochlerotatus*) *incomptus* Arnell, 1976; *Aedes* (*Ochlerotatus*) *bogotanus* Arnell, 1976; *Aedes* (*Ochlerotatus*) *deficiens* Arnell, 1976; *Aedes* (*Ochlerotatus*) *atactavittatus* Arnell, 1976; *Aedes* (*Ochlerotatus*) *synchytus* Arnell, 1976; *Aedes* (*Ochlerotatus*) *phaenotus* Arnell, 1976; *Aedes* (*Ochlerotatus*) *comitatus* Arnell, 1976 and *Aedes* (*Ochlerotatus*) *pectinatus* Arnell, 1976.

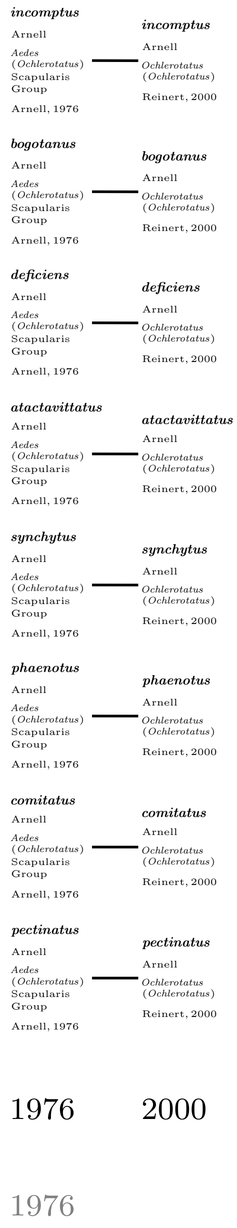

Legend: for each node, the specific and subspecific (when applicable) epithets are given in bold on top, followed by the author; afterwards follows the generic, subgeneric and informal grouping (when applicable) treatments at the time (given by the position of the node); at the bottom the reference is given for the treatment at the time; the lines represent the shifting understanding of each original combination; the x-axis gives the years for the publications, with the shadows (in gray) representing the distance of that year from the most recent year in the figure (*i.e.* 2000).
